# Measuring cancer burden in prostatic needle core biopsies: simplified assessments outperform complex measurements in assessing outcome: evidence to assist pathologist efficiency and minimize datasets

**DOI:** 10.1111/his.14886

**Published:** 2023-03-06

**Authors:** Daniel M Berney, Kier Finnegan, Kim Chu, Samson W Fine, Murali Varma, Jack Cuzick, Luis Beltran, the Transatlantic Prostate Group

**Affiliations:** ^1^ Centre for Cancer Biomarkers and Biotherapeutics Barts Cancer Institute, Queen Mary University of London London UK; ^2^ Department of Cellular Pathology Barts Health NHS Trust, The Royal London Hospital London UK; ^3^ Centre for Prevention, Detection and Diagnosis Wolfson Institute of Population Health Queen Mary University of London UK; ^4^ Department of Pathology Memorial Sloan Kettering Cancer Center New York NY USA; ^5^ Department of Cellular Pathology University Hospital of Wales Cardiff WLS UK

**Keywords:** measurement, prostate biopsy, prostate cancer, stromal gap

## Abstract

**Aims:**

The optimal method of measuring cancer extent in prostate cancer (PCa) biopsies is unknown.

**Methods and Results:**

Nine hundred eighty‐one men with clinically localised PCa managed conservatively were reviewed with follow up. The number of positive cores (NPC), the Maximum Cancer Length in a core (MCL), Total Cancer Length (TCL), and percentage of positive cores (%+cores) was calculated and univariate and multivariate analysis performed using prostate‐specific antigen (PSA), T‐stage, and Gleason score. The presence of stromal gaps (SG) was recorded. Univariate models were run where SG made a difference to the MCL.

All variables showed significant association with PCa death in univariate models. In multivariate models, incorporating PSA, T‐stage, and Gleason score, only %+cores was a significant predictor of outcome, with a 10% increase in %+cores resulting in a hazard ratio (HR) of 1.07 (likelihood‐ratio test *P* > *Χ* ^2^ = 0.01). There were 120 patients where SG made a difference to the MCL and a total of 20 events in this group. Including SG, on univariate analysis the median MCL was 10 mm and HR was 1.16 (*P* = 0.007), not including SG, the median MCL was 6 mm and HR was 1.23 (*P* = 6.3 × 10^−4^). Inclusion or exclusion of SG made no significant difference to TCL as a predictor of outcome.

**Conclusion:**

Cancer extent is a strong predictor of PCa death but only %+cores added to the multivariate model. Expressed as a fraction of NPC/total number of cores, this is the simplest method of assessment, which we favour over more complicated methods in nontargeted biopsies.

## Graphical abstract

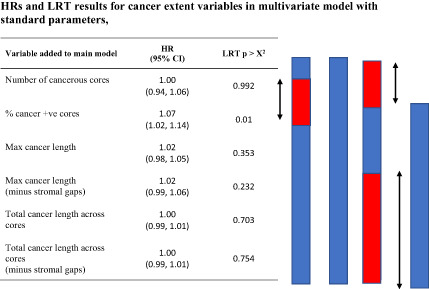

## Introduction

Histopathology biopsy data elements for prostate cancer (PCa) play a central role in risk assessment and decision making, informing initial patient management. Due to increasing screening by serum prostate‐specific antigen (PSA),^1^ and also due to rebiopsy for men on active‐surveillance programmes,^2^ the number of biopsies and cases received by genitourinary pathologists continues to rise.^3^, ^4^ Secondary to the COVID pandemic, there is also a current backlog in men requiring investigation for prostatic disease,^5^, ^6^ leading to a later surge in biopsies.^7^ Although this has been mitigated by a move to fewer systematic biopsies and more targeted biopsies in some settings,^8^ this may cause greatly increased amounts of work for many pathology departments, and prostatic pathologists in particular, for the foreseeable future.

In addition, the amount of detail required in PCa biopsy reports continues to rise. This is especially the case for assessments of disease volume on prostatic core biopsies. Prostate cancer volume has been shown by numerous groups to be a prognostic factor, although data are conflicting as to its independent significance when compared with other parameters such as the Gleason score.^9^, ^10^, ^11^

Lengths of involved cores, percentages, and detailed assessments of so‐called ‘stromal gaps’, are sometimes expected by clinical teams or performed by pathologists who use national or international datasets.^12^, ^13^, ^14^, ^15^ These assessments require considerable pathologist time, which may cause further delays in prostate biopsy reporting. It is unknown how much extra prognostic information these detailed measurements supply than more basic parameters.

We wished to explore the added value of different methods of prostatic disease volume assessments in a large cohort of men with PCa biopsies with long‐term outcome data who had been treated conservatively. Comparison of the separate techniques, it was hypothesised, would give evidence to the value they supply to clinicians for patient management.

## Methods

### The Cohort

Detailed collection methods have been described previously,^16^, ^17^ but are repeated here in brief. Men with PCa were identified from three cancer registries in the UK. Collaborating hospitals in these areas were found, and cases from these hospitals were reviewed. Men were included if they were aged <76 years at the date of diagnosis and had clinically localised PCa diagnosed by needle biopsy in 1990–2003 inclusive. The median date of diagnosis was May 2002. Patients treated with radical prostatectomy or radiation therapy within 6 months of diagnosis were excluded. Only initial hormone therapy was permitted. Those with objective evidence of metastatic disease (by bone scan, X‐ray, radiograph, computed tomography scan, magnetic resonance imaging, bone biopsy, lymph node biopsy, or pelvic lymph node dissection) or clinical indications of metastatic disease (including pathological fracture, soft‐tissue metastases, spinal compression, or bone pain), or a PSA measurement of >100 ng/ml at or within 6 months of diagnosis, were also excluded. Men who had received hormone therapy prior to the diagnostic biopsy were also excluded, because of the influence of hormone treatment on Gleason score and Grade Group. Men who died within 6 months of diagnosis or had <6 months of follow‐up were also excluded.

Original histological specimens from the diagnostic biopsies were requested. Follow‐up was conducted by use of the cancer registries, and the cutoff date was 31 December 2012. Deaths were divided into those from prostate cancer and those from other causes, according to WHO standardised criteria (WHO, 2010).

Baseline PSA level was defined as the last prediagnostic PSA measurement within 6 months before diagnosis. If no such PSA value was available, we took the first postdiagnostic PSA level within 6 months; failing that, the prediagnostic PSA level measured closest to the date of diagnosis was used. All PSA values after treatment with hormones or orchiectomy or within 3 weeks after a surgical procedure on the prostate were excluded.

National ethics approval was obtained from the Northern Multicentre Research Ethics Committee, and local ethics committee approval was obtained at each of the collaborating hospitals.

### Pathological review

A panel of three urological pathologists (D.M.B., L.B., G.S.) confirmed the diagnosis of adenocarcinoma and reassigned Grade Groups (GG) by using a contemporary and consistent interpretation of the Gleason scoring system. For every core in each case, the presence of cancer was recorded. Also, the length of each core (mm) and length of cancer (mm) were measured. In some cases, there were ‘stromal gaps’ where two malignant foci were separated by benign tissue. The length of any stromal gap was recorded, although stromal gaps were ignored if <2 mm.

**Figure 1:**
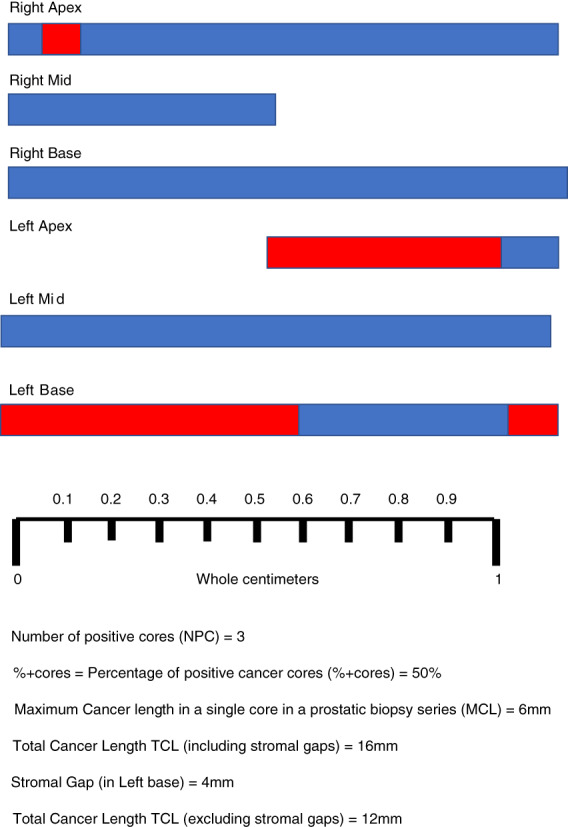
Example of different prostate biopsy cancer measurements on a prostate core set (cancer in red, benign in blue). [Color figure can be viewed at wileyonlinelibrary.com]

These data allowed calculation of the percentages of each tumour‐involved core, percentages of cores positive for cancer, and calculation of these data was performed, with and without stromal gaps. Abbreviations for these different measurements was as follows and a demonstrated example is seen in Figure 1.

Measures of cancer volume on prostatic cores.

NPC, number of positive cancer cores.

%+cores, percentage of positive cancer cores over total cancer cores.

MCL, maximum cancer length in a single core in a prostatic biopsy series (mm).

TCL, total cancer length: addition of all cancer core lengths in a biopsy series (mm).

SG, stromal gap (mm).

The panel met and discussed all controversial cases and a selection of others to audit the dataset. In keeping with the ISUP 2014 recommendations and the grading in World Health Organization (WHO) 2016, cribriform and glomeruloid glands were all assigned Gleason pattern 4. Analysis of this cohort with regard to pattern and grade of disease has been published previously.^17^

### Statistical Analysis

Survival analysis was performed with a Cox proportional hazards model. The primary endpoint was time to death from prostate cancer. All events were used for estimation of hazard ratios (HRs), and observations were censored on the date of last follow‐up or death from other causes. All events were used for estimation of HRs (maximum follow‐up 232 months), but follow‐up was censored at 10 years for prediction of 10‐year risks. HRs for continuous variables were calculated from the interquartile range. Those of ordinal variables were calculated by an increase of 1 (Gleason score) or by 10% in number (biopsy number), as appropriate.

Extent of disease was measured in each core by the methods described above. Covariates included in the statistical analysis were Gleason scores by overall grade. This had been shown to be comparable to ‘worst’ grade in a previous article.^18^

The PSA level was modelled as the natural logarithm of [1 + PSA (ng/ml)]. Patients with values of >100 ng/ml were excluded as likely to have metastatic disease. Missing PSA values were imputed by use of a median regression with GG, age, and extent of disease as predictors, and PSA as outcome. Missing T‐stage values were imputed using the median clinical T‐stage among all patients.

A univariate model was applied to Gleason score, baseline PSA level, T‐stage, and the multiple different methods of tumour volume measurement. A multivariate Cox proportional hazard model applied performed Gleason score, baseline PSA level, extent of disease, and T‐stage. The primary event of interest was time to death from prostate cancer. A stepwise model selection was performed.

Spearman's rank correlation was estimated between all variables. All applied tests were two‐sided, and *P* < 0.05 were accepted as statistically significant. No *P*‐value adjustment was performed for multiple comparisons. Statistical analyses were performed with r (R Core Team 2018. R: A language and environment for statistical computing. R Foundation for Statistical Computing, Vienna, Austria. http://www.R‐project.org/).

## Results

The data included a cohort of 981 men in which there were 204 prostate cancer deaths in the study period. Three baseline PSA values were missing and were imputed using linear regression. Disease stage at baseline was missing for 228 men and were imputed using multinomial logistic regression. An overview of the Gleason score, Grade Group, and number of biopsies taken in each case is given in Table S1.

Univariate Cox proportional hazards models were run to assess the ability of the cancer extent measurements to predict mortality. Gleason score, PSA, and T‐stage were also included in these models.

All the methods of assessment of tumour volume were highly predictive of prostate cancer death, but all were inferior hazard ratios to the standard parameters of Gleason score, serum PSA, and T‐stage (Table 1).

**Table 1:** Univariate model for baseline parameters and measures of tumour extent

Variable	Median (IQR) [min, max]	HR (95% CI)	*P*‐value	Harrell's c‐statistic*
Gleason score	7 (6, 7) [6, 10]	1.97 (1.74–2.23)	9.3 × 10^−28^	0.692
Log (PSA + 1)^†^	14.1 (8.1, 31) [0.1, 100]	2.13 (1.80–2.52)	1.1 × 10^−18^	0.683
Clinical T‐stage^‡^	2 (2, 2) [1, 3]	3.16 (2.46–4.06)	1.8 × 10^−19^	0.661
*N* cancerous cores	3 (1, 4) [1, 13]	1.16 (1.09–1.22)	1.8 × 10^−7^	0.617
% cancer +ve cores^§^	50 (25, 83) [6, 100]	1.23 (1.17–1.29)	2.8 × 10^−18^	0.690
Max cancer length (mm)	7 (3, 11) [0.4, 25]	1.11 (1.08–1.14)	5.0 × 10^−14^	0.648
Max cancer length (minus stromal gaps) (mm)	7 (3, 11) [0.4, 25]	1.12 (1.09–1.15)	1.6 × 10^−15^	0.658
Total cancer length (mm)	12 (4, 31) [0.5, 136]	1.02 (1.01–1.02)	5.3 × 10^−11^	0.655
Total cancer length (minus stromal gaps) (mm)	11 (4, 30) [0.5, 132]	1.02 (1.01–1.02)	1.5 × 10^−11^	0.661

*N* = 981.

* Measured from 0.5 to 1, where 0.5 implies model prediction is no better than chance, 1 implies perfect prediction.

^†^ Three values were imputed (as in previous analyses). Log (PSA + 1) used in the models.

^‡^ 228 values were imputed (as in previous analyses).

^§^ HR per 10% increase in proportion of cancerous cores.

Multivariate proportional hazards models were run to see which predictors were most correlated with cancer mortality. As well‐established predictors of cancer mortality, Gleason score, log‐PSA, and T‐stage were used as predictors in a ‘main’ multivariate model (Harrell's c‐statistic = 0.743), with cancer extent measures being added to the model to determine how informative they were in the presence of other variables. All are highly significant, with hazard ratios of ~1.5 (Table 2).

**Table 2:** Main multivariate model for standard parameters

Variable	HR (95% CI)	*P*‐value
Gleason score	1.54 (1.32–1.79)	2.2 × 10^−8^
Log(PSA + 1)	1.53 (1.27–1.86	9.8 × 10^−6^
Clinical T‐stage	1.58 (1.18–2.12)	2.0 × 10^−3^

Harrell's c‐statistic = 0.743.

Six measurements of cancer extent were added to this model in order to assess their contribution in predicting cancer mortality. The hazard ratio reported for the percentage of cancerous cores was adjusted to reflect a 10% increase. A likelihood ratio test was carried out to determine how informative the variable was alongside the variables in the main model. The percentage of cancerous cores was a significant predictor of cancer mortality in both the univariate and multivariate models, with a 10% increase in percentage cancerous cores resulting in an HR of 1.07 when added to the main model in multivariate analysis. No other variable added any information to the multivariate model (Table 3).

**Table 3:** Hazard ratios and LRT results for cancer extent variables in multivariate model

Variable added to main model	HR (95% CI)	LRT *P* > *Χ* ^2^
Number of cancerous cores	1.00 (0.94–1.06)	0.992
% cancer +ve cores	1.07 (1.02–1.14)	0.01
Max cancer length	1.02 (0.98–1.05)	0.353
Max cancer length (minus stromal gaps)	1.02 (0.99–1.06)	0.232
Total cancer length across cores	1.00 (0.99–1.01)	0.703
Total cancer length across cores (minus stromal gaps)	1.00 (0.99–1.01)	0.754

LRT, Likelihood‐ratio test.

For 861/981 patients, the calculated maximum cancer length per‐patient, across cores, was the same regardless of whether or not stromal gaps were included in the calculation. Univariate models were run on the 120 patients where stromal gaps made a difference to the max cancer length (Table 4). There were 20 prostate cancer deaths in this cohort. All measures of cancer extent were significant. The characteristics of this cohort are shown in Table S2.

**Table 4:** Univariate model results for 120 patients with stromal gaps present

Variable	Median (IQR) [min, max]	HR (95% CI)	*P*‐value	Harrell's c‐statistic*
Gleason score	7 (6, 7) [6, 9]	2.67 (1.64–4.33)	7.3 × 10^−5^	0.707
Log(PSA + 1)	15.4 (9,32) [0.4, 95]	2.79 (1.49–5.20)	0.001	0.674
Clinical T‐stage	2 (2, 2) [1, 3]	3.42 (1.45–8.07)	0.005	0.656
*N* cancerous cores	3 (2, 5) [1, 12]	1.20 (1.01–1.42)	0.034	0.661
% cancer +ve cores^†^	56 (33, 83) [6.7, 100]	1.35 (1.13–1.63)	0.001	0.742
Max cancer length	10 (6, 12) [2, 20]	1.16 (1.04–1.30)	0.007	0.656
Max cancer length (minus stromal gaps)	6 (3, 9) [1, 19]	1.23 (1.09–1.38)	6.3 × 10^−4^	0.698
Total cancer length	16 (8, 31) [2, 102]	1.02 (1.00–1.04)	0.039	0.697
Total cancer length (minus stromal gaps)	12 (5, 26) [1, 94]	1.02 (1.00–1.04)	0.027	0.717

*N* = 120.

* Measured from 0.5 to 1, where 0.5 implies model prediction is no better than chance, 1 implies perfect prediction.

^†^ HR per 10% increase in proportion of cancerous cores.

Clinical T‐stage was not significant in the multivariate analysis of this patient subset (*P* = 0.53) and so not included in this and was not included in this multivariate model (Table 5).

**Table 5:** Main multivariate model for 120 patients with stromal gaps present

Variable	HR (95% CI)	*P*‐value
Gleason score	2.17 (1.27–3.71)	0.005
Log(PSA + 1)	2.13 (1.11–4.07)	0.023

Harrell's c‐statistic = 0.746.

With these limited numbers of patients, no measures on cancer extent reached clinical significance on the multivariate model (Table 6). To compare the maximum tumour length with or without stromal gaps, a likelihood ratio test was carried out to determine the impact of adding the other variables into the model. The results (Table S3) indicate that MCL− offered more information to the model than MCL+.

**Table 6:** Hazard ratios and LRT results for cancer extent variables in multivariate model for 120 patients with stromal gaps present

Variable added to main model	HR (95% CI)	LRT *P* > *Χ* ^2^
Number of cancer +ve cores	1.07 (0.88–1.29)	0.54
% cancer +ve cores	1.19 (0.95–1.49)	0.13
Max cancer length	1.04 (0.92–1.18)	0.53
Max cancer length (minus stromal gap)	1.10 (0.95–1.27)	0.19
Total cancer length across cores	1.00 (0.98–1.02)	0.90
Total cancer length across cores (minus stromal gap)	1.00 (0.97–1.02)	0.89

LRT, Likelihood‐ratio test.

## Discussion

There has been an ongoing debate about the optimal method of measuring cancer extent in prostatic biopsies. In 2008, a systematic review demonstrated the paucity of good evidence, with most articles relying on pathological surrogates for outcome rather than outcome measures.^19^ Since that time a number of datasets have been published that have tended to increase the number of options and methodologies for cancer measurements, with no consensus on the optimal choice. The Royal College of Pathologists dataset^14^ suggests that ‘pathologists should report the number of cores involved and at least one of the methods of estimating tumour extent’.

The ICCR dataset suggests that as well as reporting positive cores, linear extent should be reported as either millimetres cancer length or % cancer in each core or as a composite measure of cancer involvement in all cores.^12^

The College of American Pathologists have most recently updated their guidance in 2021. They suggest ‘the number of positive cores out of the total number of cores should always be reported, except in situations where fragmentation precludes accurate counting. The estimated percentage of prostatic tissue involved by tumour and/or the linear millimeters of the tumour should also be reported. Reporting of the positive core with the greatest percentage of tumor is an option since in some active surveillance (AS) protocols, the presence of any cores with >50% involvement is an exclusion criterion’.^15^

All three protocols try to tackle the issue of stromal gaps, and acknowledge the evidence lack. It has been suggested that most discontinuous foci are from the same tumour after radical prostatectomy have been examined.^20^, ^21^

Supporting this, one study concentrating on low‐risk prostate cancers showed that including stromal gaps in cancer measurements correlated better with pathological surrogates for outcome on radical prostatectomy.^22^

The argument against inclusion of stromal gaps relates to the situation where it might preclude a patient from the option of active surveillance^23^; for instance, where 1 mm cancer foci at either end of a core are separated by a large stromal gap, meaning that a measurement might be large enough to preclude surveillance.

We have previously performed a study on an earlier cohort of prostate cancers. To our knowledge, this was the only previous assessment of tumour burden in a conservatively treated cohort which had death from prostate cancer as an outcome.^24^ Although the findings from that study showed similar findings with the percentage of cores with cancer being marginally the strongest predictor, the number of cores taken in that cohort were often limited and no assessment of stromal gaps was undertaken.

The evidence base on which to assess these findings and correlate with disease volumes is often lacking. Although biopsy disease burdens on biopsy can be directly compared with the radical prostatectomy specimens from patients, this is a highly selected subset of biopsy specimens, excluding patients who received other modalities of radical therapy or active surveillance.

A prostatic biopsy remains a sample of the prostatic tissue, and thus is prone to considerable error in sampling, which is unavoidable.

Our data demonstrates that in this cohort of conservatively managed patients, simpler methods, especially the percentage of positive cores, add more predictive value for prostate cancer death than more complex, time‐consuming ones. These data may also render the ongoing debate on inclusion and exclusion of ‘stromal gaps’ somewhat redundant.

The area in which detailed measurements may still be helpful, in our opinion, is in candidates for active surveillance, where lengths of cancer per core may influence active surveillance decisions.^23^ However, in higher‐grade tumours from Grade Groups 2–5, as opposed to measures of pattern 4/5, we suggest that other detailed measurements add little to the decision‐making process and in biopsy material simplified techniques could be used, potentially saving considerable pathologist time.

In the future, we acknowledge that the advent of artificial intelligence algorithms applied to cancer foci may be a great aid in measuring these parameters,^25^, ^26^ although such models to date have primarily concentrated on PCa grading. We would contend that due to the variable nature of the biopsy procedure, it would remain of indefinite significance in prognostic independence and choice of treatment method.

The strengths of this cohort are its size, long‐term follow‐up, detailed pathological review with modern criteria, and choice of a clinical, rather than pathologic (radical prostatectomy findings), endpoint.

The main weaknesses of the cohort are its retrospective nature, the relatively fewer number of cores compared to modern practice, and due to the focus on long‐term follow‐up, the lack of targeted biopsy and mpMRI data.

In summary, we have demonstrated the power of simpler techniques in calculating prostatic disease volume in biopsy material. Our data suggest that national and international datasets may be simplified to save considerable effort, as prostate cancer remains a significant health burden in many countries.

## Author contributions

D. M. Berney, L. Beltran reviewed the pathological material. K. Finnegan and K. Chu performed the statistical analysis. D. Berney wrote the article and guided this particular TAPG article with assistance from M. Varma and S. Fine who provided intellectual input. J. Cuzick leads the TAPG consortium and initiated the whole TAPG study. All authors reviewed and commented upon the article.

## Funding information

We gratefully acknowledge support from Cancer Research UK, ORCHID, a SPORE grant from the US National Cancer Institute (P50CA09629), the David H. Koch Fund, and Myriad Genetics.

## Supporting information


**Table S1.** Characteristics of cohort including distribution of Gleason scores, Grade groups and cT stage.

**Table S2.** Details of 120 cases where the stromal gaps were identified and affected the Maximum cancer length measurements.

**Table S3.** Comparison of predictive value of maximum cancer length with/without stromal gaps.

## Data Availability

Full Data on the TAPG cohort is held by Prof J Cuzick and will be shared on reasonable request.
