# Supplementary material for: Measuring cancer burden in prostatic needle core biopsies: simplified assessments outperform complex measurements in assessing outcome: evidence to assist pathologist efficiency and minimize datasets
Source: Histopathology. 2023 Mar 6;82(7):1021–8. doi: 10.1111/his.14886 (PMC10192044; doi:10.1111/his.14886)
Supplement: Supplementary file 1 — Table S1. Characteristics of cohort including distribution of Gleason scores, Grade groups and cT stage. [file HIS-82-1021-s001.docx]

Supplemental table 1.

Characteristics of cohort including distribution of Gleason scores, Grade groups and cT stage

| **Gleason score** | **Number of men** | **Percentage** |
| --- | --- | --- |
| 6 | 303 | 30.9 |
| 7 | 510 | 52.0 |
| 8 | 56 | 5.7 |
| 9 | 101 | 10.3 |
| 10 | 11 | 1.1 |

| **Grade Group** | **Number of men** | **Percentage** |
| --- | --- | --- |
| 1 | 303 | 30.9 |
| 2 | 301 | 30.7 |
| 3 | 209 | 21.3 |
| 4 | 56 | 5.7 |
| 5 | 112 | 11.4 |

**cT-stages**

| **cT-stages** | **Number of men** | **Percentage** |
| --- | --- | --- |
| 1 | 136 | 13.9 |
| 2 | 670 | 68.3 |
| 3 | 175 | 17.8 |

|  | **Median (IQR) [min,max]** |
| --- | --- |
| **Number of biopsies** | 6 (5, 8) [1, 19] |
