# Supplementary material for: Measuring cancer burden in prostatic needle core biopsies: simplified assessments outperform complex measurements in assessing outcome: evidence to assist pathologist efficiency and minimize datasets
Source: Histopathology. 2023 Mar 6;82(7):1021–8. doi: 10.1111/his.14886 (PMC10192044; doi:10.1111/his.14886)
Supplement: Supplementary file 2 — Table S2. Details of 120 cases where the stromal gaps were identified and affected the Maximum cancer length measurements. [file HIS-82-1021-s003.docx]

Supplemental Table 2.

Details of 120 cases where the stromal gaps were identified and affected the Maximum cancer length measurements.

| **Variable** | **Median (IQR)[min,max]** | **N (%)** |
| --- | --- | --- |
| Gleason score | 7 (6, 7) [6, 9] | 120 (100.00) |
| 6 | - | 33 (27.50) |
| 3+4 | - | 44 (36.67) |
| 4+3 | - | 35 (29.17) |
| 8 | - | 3 (2.50) |
| 9 | - | 5 (4.17) |
| Log(PSA + 1)^a^ | 15.4  (9.0, 31.9) [0.4, 95.0] | 120 (100.00) |
| Clinical T-stage^b^ | 2  (2, 2) [1, 3] | 120 (100.00) |
| 1 | - | 14 (11.67) |
| 2 | - | 89 (74.17) |
| 3 | - | 17 (14.17) |
| N cancerous cores | 3  (2, 5) [1, 12] | 120 (100.00) |
| % cancer +ve cores | 56  (33, 83) [7, 100] | 120 (100.00) |
| Max cancer length (mm) | 10  (6, 12) [2, 20] | 120 (100.00) |
| Max cancer length (minus stromal gaps) (mm) | 6  (3, 9) [1, 19] | 120 (100.00) |
| Total cancer length (mm) | 16  (8, 31) [2, 102] | 120 (100.00) |
| Total cancer length (minus stromal gaps) (mm) | 11.5  (5, 26) [1, 93.5] | 120 (100.00) |
| N cores taken | 6  (5, 8) [1, 15] | 120 (100.00) |

N = 120

^a^ 1 values were imputed (as in previous analyses). Log (PSA + 1) used in the models;

^b^ 22 values were imputed (as in previous analyses)
