# Supplementary material for: Measuring cancer burden in prostatic needle core biopsies: simplified assessments outperform complex measurements in assessing outcome: evidence to assist pathologist efficiency and minimize datasets
Source: Histopathology. 2023 Mar 6;82(7):1021–8. doi: 10.1111/his.14886 (PMC10192044; doi:10.1111/his.14886)
Supplement: Supplementary file 3 — Table S3. Comparison of predictive value of maximum cancer length with/without stromal gaps. [file HIS-82-1021-s002.docx]

### Supplemental Table 3 : Comparison of predictive value of maximum cancer length with/without stromal gaps

| **Variable** | **n** | **Median (IQR) [min, max]** | **HR  (95% CI)^a^** | **p-value** | **Harrell’s  c-statistic** | **LRT** | **^b^P >Χ^2^** |
| --- | --- | --- | --- | --- | --- | --- | --- |
| MCL including stromal gaps | 120 | 10  (6, 12) [2, 20] | 1.16  (1.04, 1.30) | 6.6x10^-4^ | 0.656 | 0.698 | 0.403 |
| MCL  minus stromal gaps | 120 | 6  (3, 9) [1, 19] | 1.23 (1.09, 1.38) | 6.3x10^-4^ | 0.698 | 5.200 | 0.023 |
| ^a^ Hazard ratios from univariate models  ^b^ Likelihood ratio test comparing multivariate model (with and without stromal gaps) | | | | | | | |
